# Subphenotypes in acute kidney injury: a narrative review

**DOI:** 10.1186/s13054-022-04121-x

**Published:** 2022-08-19

**Authors:** Suvi T. Vaara, Pavan K. Bhatraju, Natalja L. Stanski, Blaithin A. McMahon, Kathleen Liu, Michael Joannidis, Sean M. Bagshaw

**Affiliations:** 1 Division of Intensive Care Medicine, Department of Anesthesiology, Intensive Care and Pain Medicine, Meilahti Hospital, University of Helsinki and Helsinki University Hospital, PO Box 340, 00290 Helsinki, Finland; 2 Division of Pulmonary, Critical Care and Sleep Medicine, University of Washington, Seattle, USA; 3 Sepsis Center of Research Excellence (SCORE), University of Washington, Seattle, USA; 4 Division of Critical Care Medicine, Department of Pediatrics, Cincinnati Children’s Hospital Medical Center, University of Cincinnati College of Medicine, Cincinnati, USA; 5 Division of Nephrology, Department of Medicine, Medical University of South Carolina, Charleston, SC USA; 6 Divisions of Nephrology and Critical Care, Departments of Medicine and Anesthesia, University of California, San Francisco, USA; 7 Division of Intensive Care and Emergency Medicine, Department of Internal Medicine, Medical University of Innsbruck, Innsbruck, Austria; 8 Department of Critical Care Medicine, Faculty of Medicine and Dentistry, University of Alberta and Alberta Health Services, Edmonton, Canada

**Keywords:** Acute kidney injury, Biomarkers, Critically ill, Heterogeneity, Latent class analysis, Subphenotypes

## Abstract

Acute kidney injury (AKI) is a frequently encountered syndrome especially among the critically ill. Current diagnosis of AKI is based on acute deterioration of kidney function, indicated by an increase in creatinine and/or reduced urine output. However, this syndromic definition encompasses a wide variety of distinct clinical features, varying pathophysiology, etiology and risk factors, and finally very different short- and long-term outcomes. Lumping all AKI together may conceal unique pathophysiologic processes specific to certain AKI populations, and discovering these AKI subphenotypes might help to develop targeted therapies tackling unique pathophysiological processes. In this review, we discuss the concept of AKI subphenotypes, current knowledge regarding both clinical and biomarker-driven subphenotypes, interplay with AKI subphenotypes and other ICU syndromes, and potential future and clinical implications.

## Background

Acute kidney injury (AKI) is a common syndrome in hospitalized populations and especially in the critically ill [1, 2]. It is associated with prolonged hospitalization, receipt of kidney replacement therapy (KRT), persistent loss of kidney function, and death [1–3]. AKI is diagnosed based on clinical features indicating the deterioration of kidney function, namely increased level of serum creatinine and/or decreased urine output [4].

While the current definition of AKI has enhanced clinical recognition of AKI and promoted critical concepts applicable to AKI populations, combining all patients with AKI into one group may hide sub-groups that are more tightly linked to clinical outcomes [5] and conceal unique pathophysiologic processes specific to certain AKI populations [6]. Supporting this notion, multiple research groups have shown that diversity within the AKI clinical syndrome exists and a ‘one size fits all’ approach may not be ideal [7–10]. Thus, existing heterogeneity within the group of AKI patients may explain why multiple clinical trials have yet to identify effective pharmacotherapy for its prevention or treatment [3, 4, 11]. Furthermore, the efficacy of certain already tested pharmacotherapies may have been concealed by the existing heterogeneity in the trial population and lack of suitable measures to detect improved outcomes [12, 13].

This review aims to describe the concept of subphenotypes in AKI, current knowledge regarding both clinical and biomarker-driven subphenotypes, interplay with the subphenotypes with other ICU syndromes such as acute respiratory distress syndrome (ARDS), and potential future and clinical implications.

## Concept of subphenotypes

**Fig. 1:**
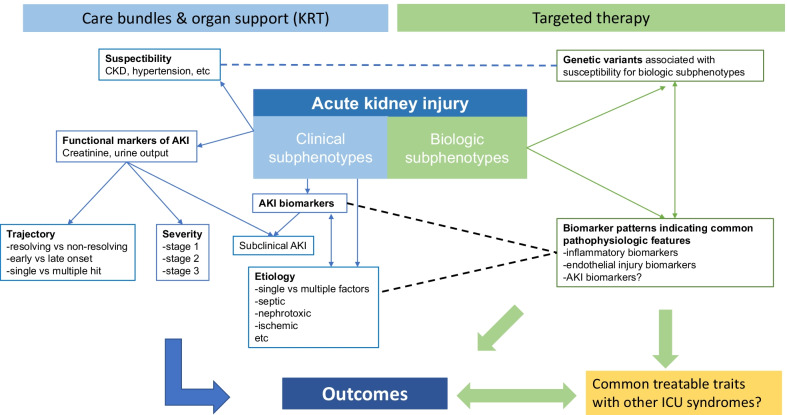
Subphenotypes in acute kidney injury. AKI; acute kidney injury, CKD; chronic kidney disease, ICU; intensive care unit, KRT; kidney replacement therapy

Among critically ill patients, several syndromic diagnoses (or phenotypes) are recognized, such as AKI [4], ARDS [14], sepsis [15], and delirium. These diagnoses encompass a wide variety of distinct clinical features, varying pathophysiology, etiology, risk factors and clinical course, and finally, very different short- and long-term outcomes. A subphenotype is a distinct group of patients within a phenotype such as AKI who share common features, risk factors, biomarker positivity, response to treatment, or outcomes that separates this subphenotype from other groups of patients within the phenotype [16]. Thus, multiple ways to classify patients into subphenotypes exist (Fig. 1). Severity scoring according to clinical features (such as magnitude of creatinine rise) into subgroups of differing outcomes (such as stage 1 to 3 AKI) [4] has a long tradition in daily clinical practice. However, classifying patients using multiple clinical variables and biomarkers to more specific biologic subphenotypes may better reflect the underlying pathophysiology, facilitate customized approaches to care, and ultimately find targeted therapies.

**Fig. 2:**
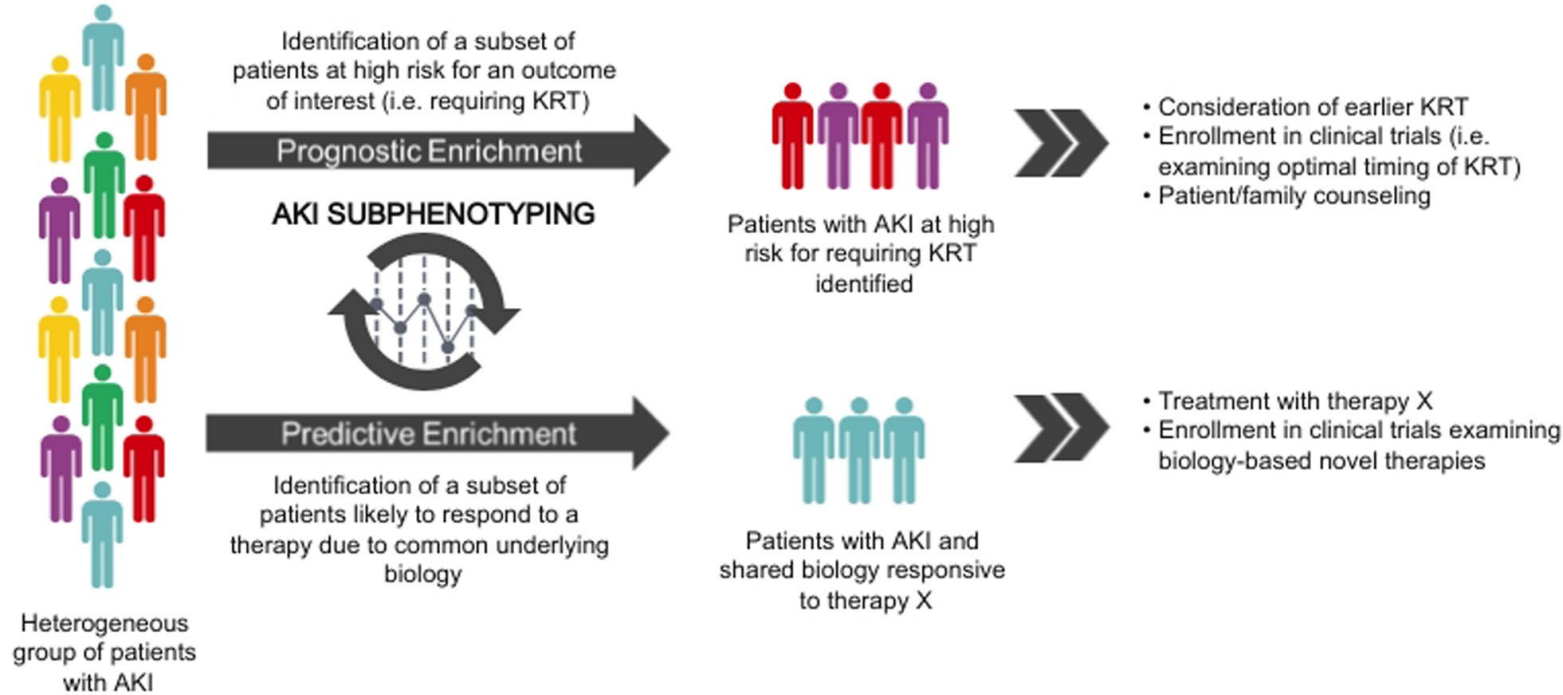
Concept of prognostic and predictive enrichment**.** AKI; acute kidney injury, KRT; kidney replacement therapy

Regardless of the strategy used to subphenotype AKI, the overarching goal should remain the same: to cohort patients into groups with unique prognostic and/or therapeutic implications [17, 18]. Subgrouping patients in this manner is termed *enrichment*, a central tenet of precision medicine. A general schematic of how subphenotyping can facilitate *prognostic enrichment* (i.e. identifying patients likely to have a disease-related outcome of interest) and *predictive enrichment* (i.e. selecting patients more likely to respond to a given therapy on the basis of biology) to personalize AKI management is shown in Fig. 2.

## Methodological aspects

Relatively novel methods to find subphenotypes within phenotypes include clustering methods such as latent class analysis (LCA) and k-means clustering. LCA is a frequently used mixture model that presumes that an unobserved categorical variable exists that classifies the heterogeneous population into mutually exclusive latent classes (homogeneous subgroups) [19]. Observed variables are used to predict the membership of these unobserved or latent groups [19]. As in other types of statistical models, selection of the variables for the model should be carefully considered and be based on the research question. From the fitted LCA model, probabilities of class membership are generated that can then be used to assign patients to latent classes [20]. The number of classes is selected on the basis of the best model with the fewest number of classes using various criteria [20]. Exploring the variables that come up in the process as the strongest definers of the latent classes can provide useful hints of the potential pathophysiologic background.

The current methods also have some limitations. First, one must remember that the selection of variables has been made bearing the research question and study hypothesis in mind, and most subphenotype analyses thus far have been conducted using existing databases or clinical trial datasets that have a limited selection of variables available. This has also generated heterogeneity in the identified subphenotypes. Therefore, besides further validation of the discovered subphenotypes, prospective studies aimed at detecting subphenotypes reflecting the hypothesized pathophysiology would help to find the best combination variables to define the novel subphenotypes. Second, as clustering analysis is a powerful tool of finding distinct groups, the results of such analyses must be carefully interpreted to avoid over-optimistic conclusions of finding something that may not actually exist or be replicated in other studies. Thus, a good starting point would be a study protocol that is based on sound pathophysiologic hypotheses. The protocol should also include the development of clinically feasible, rapid method to identify subphenotypes that may include for example, a novel combination of routinely measured parameters with a point-of-care measurement of a biomarker. Obviously, even before subphenotype-directed therapeutic randomized trials, enormous work is needed that is not possible without international collaboration.

## Clinical subphenotypes of AKI

Currently, the defining criteria for AKI are based purely on the absolute or relative increase in serum creatinine or a decrease in urinary output [4]. Therefore, the definition of AKI does not include information about the trajectory of AKI, AKI biomarkers, or renal recovery criteria. Moreover, it does not acknowledge the significance of recurrent AKI ‘hits’ within a single hospitalization. However, AKI is heterogeneous in its etiology. Single AKI episodes can differ based on timing of injury, rate of AKI development, natural history specific to etiology, prediction of clinical outcome, and finally, severity. Additionally, outcomes are influenced by baseline kidney function, the duration of AKI, and the interaction with non-kidney organ injury and dysfunction [4]. Moreover, the variability in the application of KDIGO criteria is a great source of heterogeneity and reported varying outcomes especially in database and registry-related research [21].

**Fig. 3:**
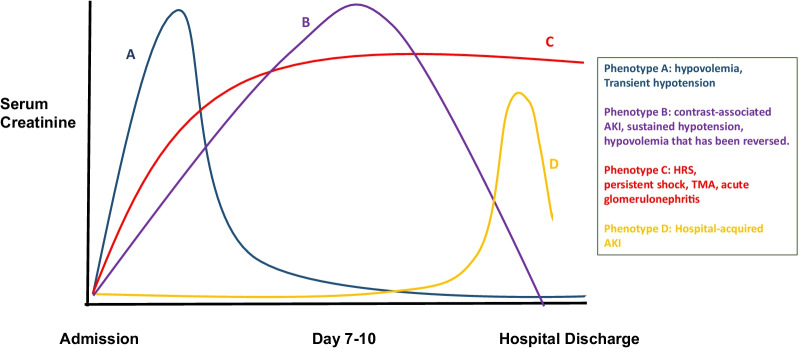
Scenarios presenting phenotypes based on the etiology or trajectory of acute kidney injury. AKI; acute kidney injury, HRS; hepatorenal syndrome, TMA; thrombotic microangiopathy

The concept of pre-renal, intrinsic, and post-renal AKI has a long tradition to stratify the etiology of AKI and is among the oldest ways to subphenotype AKI. As the diagnosis of AKI does not account for etiology, conventional diagnostic tools may reveal disease processes with available specific treatment, such as thrombotic microangiopathies, glomerulonephritis, or post-renal obstruction. In addition to these, using creatinine trajectories allow a more tailored and immediate approach to management (Fig. 3).

### Creatinine-based AKI trajectories

Subphenotypes of AKI can be identified from functional changes including creatinine trajectories during the AKI diagnosis (Table 1). In a large observational study, authors identified resolving and non-resolving AKI subphenotypes based on the early trajectory of creatinine values in ICU patients using single creatinine changing trend to model the probability of death [7]. Another study by Guitterez et al. described the trajectory of creatinine rise analyzed in a longitudinal fashion after exposure to radiocontrast media in 98 subjects who underwent cardiac catheterization [22]. The authors used a random intercept and slope model to describe the trajectory of creatinine rise limited to an observational time of 5.5 ± 5.1 days. However, generalizability of model trajectory was not validated in other cohorts of patients with higher rates of AKI. Neither of these two studies used detailed clinical data to identify AKI trajectory subphenotypes. A more recent study of 5,294 post cardiopulmonary bypass patients divided into a development and a validation cohort, identified 12 novel AKI trajectory subphenotypes with distinct postoperative serum creatinine trajectories over time [23]. In this study, the authors used latent class mixed modeling to identify several other features, including patient and procedural characteristics, post-operative complications, and long-term outcome data. Notably, four high-risk phenotypes had greater long-term risk for death relative to lower risk classes.

**Table 1:** Summary of Studies that Have Described (A) Serum Creatinine Trajectory Trends during AKI (B) the duration of AKI recovery and poor renal outcomes

Author	Time period	AKI definition	Data source	Data analysis method	n, patients	Clinical setting	Reported SCr trajectory	Definition of mortality/adverse renal outcomes
A								
Guitterezet al. [22]	NA	Rise in serum creatinine > 25% above baseline	Single US center	Random intercept and slope model	98	Cardiac catherization	Maximum creatinine within 5.5 days ± 5.1 days	NA
Bhatraju et al. [7]	2003–2005	KDIGO SCr criteria	Single and multi- Center US	Spaghetti plots	1,914 and 1,867	ICU admissions	AKI during the first 72 h in the ICU	Death prior to hospital discharge
Smith et al. [24]	2009–2017	KDIGO SCr criteria	(NHLBI) ARDS Network and Single US center	Dynamic time-warping, the Bray–Curtis dissimilarity and qgglomerative hierarchical clustering	6,816	ICU admissions	AKI during the first 7 days in the ICU	Death prior to hospital discharge
Andrew et al. [23]	2000–2009	KDIGO SCr criteria	Single US center	Latent class mixed modeling	2,647 and 2,647	Cardiac Surgery	AKI experienced within the preoperative and first four postoperative days	Death 3 years postoperatively
B								
Ozrazgat-Baslanti et al. [25]	2012- 2019	KDIGO SCr criteria	Single US center	Propensity score-based inverse weighting and Kaplan–Meier curves	156, 699	All hospitalizations	Classified (no AKI, rapidly reversed AKI, persistent AKI with and without renal recovery)	1, 3- year mortality, need for new RRT, new CKD within 90 days or 1-year, CKD progression
Siew et al. [26]	2002–2014	KDIGO stages 2 to 3 AKI	US Veterans	Multivariable Cox proportional hazards regression	47,903	All hospitalizations	AKI recovery to within 120% of baseline SCr level within 90 days	Sustained 40% decline in eGFR from closest SCr
Bhatraju et al. [27]	2009–2015	Modified KDIGO	Multi-center US ASSESS-AKI study	Cox proportional hazards regression	1,538	All hospitalizations	AKI resolving with 72 h of AKI diagnosis	Major adverse kidney events

*AKI* Acute kidney injury, *ARDS* Acute respiratory distress syndrome, *CKD* Chronic kidney disease, *ICU* Intensive care unit, *KDIGO* Kidney diseases improving global outcomes, *SCr* serum creatinine

**Fig. 4:**
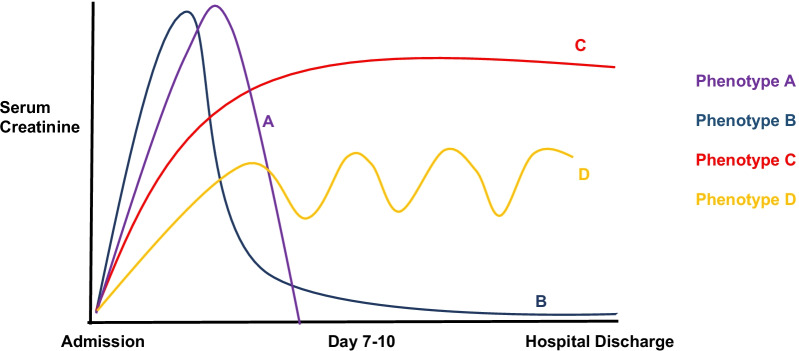
Hypothetical patient scenarios with different AKI recovery subphenotypes and influence on renal outcomes

A meticulous study by Smith et al. [24] used a population-based approach to align and compare long and short KDIGO trajectories. Additionally, they used clinical-oriented approach to determine the number of AKI trajectory subphenotypes including the identification of a critical AKI trajectory feature. They assessed 6,816 ICU patients that developed any stage of KDIGO AKI with this model and found that the trend or shape of trajectory appeared to be more associated with inpatient mortality rates rather than the maximum KDIGO stage. As shown in Fig. 4 with hypothetical patient scenarios, patients with a lower maximum KDIGO stage and a gradual decline in kidney function over time (subphenotype C & D) had a higher ICU mortality rate when compared to those patients who had a high maximum KDIGO score on arrival in the ICU but rapidly recovered by day 3 (subphenotype A & B).

### Severity and delayed resolution of AKI

The identification of subgroups of patients with AKI based on the trajectory of kidney function recovery after an AKI episode is also a potentially important and clinically intuitive parameter for risk stratification of AKI. In a large cohort of 47,903 adult US veteran patients, patients were subgrouped by the duration of time from peak serum creatinine to recovery of kidney function [26]. The primary outcome was a sustained 40% decline in estimated glomerular filtration rate or kidney failure [26]. Patients with a protracted AKI recovery course were at higher risk for earlier loss of kidney function after recovery was complete [26]. This study was corroborated further in a retrospective longitudinal cohort study of 156,699 hospitalized, ICU and non-ICU patients focused on persistent AKI [25]. AKI was classified as rapidly reversed AKI or persistent AKI (AKI lasting longer than 48 h with and without renal recovery) and compared to individuals with no AKI. Persistent AKI without renal recovery was associated with approximately fivefold increased hazard rates for all-cause mortality compared with no AKI in the full cohort as well as in the ICU and non-ICU subcohorts, independent of AKI severity [25].

Furthermore, functional tests can be used to identify patients who are likely to be AKI non-resolvers. The furosemide stress test [28] assesses the urine output response to a large single dose of intravenous furosemide. Patients with a poor response have been found to have a higher likelihood of progression of AKI [29]. It has been used as a method to stratify patients for intervention studies [30].

## Biomarker-driven AKI subphenotypes

A number of blood and urinary biomarkers have been shown to predict the development of AKI, such as plasma or urinary neutrophil gelatinase-associated lipocalin (NGAL), urinary kidney injury molecule 1 (KIM-1), urinary tissue inhibitor of metalloproteinase-2 (TIMP-2), and insulin-like growth factor-binding protein 7 (IGFBP7), and others [31]. Biomarkers have also been used to identify AKI subphenotypes. One such biomarker-derived AKI subphenotype is subclinical AKI. Subclinical AKI refers to a clinical condition where structural kidney injury occurs without a rise in creatinine. Studies have shown that elevations in urinary NGAL or KIM-1 without a rise in creatinine subsequently predicted initiation of KRT or in-hospital mortality [32, 33]. In a cardiac surgery cohort, elevations in urinary interleukin-18 and KIM-1 were independently associated with higher 3-year mortality in those patients without AKI [34]. More recently, alternative plasma and urinary biomarkers have again demonstrated a stepwise increase in adverse outcomes in those with subclinical AKI compared with patients with established AKI [35, 36]. This association underlines the problem with creatinine, namely kidney damage with association to patient-centered outcomes may occur even without an evident creatinine increase. Whether such damage is limited to the kidneys as the kidney-specific biomarkers imply, or a problem encompassing also other organs such as endothelium, needs to be further elucidated.

Biomarkers have also been found to predict the non-recovery of AKI. In an analysis comprising 331 patients, C–C motif chemokine ligand 14 (CCL14) was discovered to stratify patients according to the likelihood of persistent severe AKI [37]. These results imply that in future, if validated, biomarkers could help to allocate patients into both trials searching for methods to enhance AKI recovery and to ensure adequate follow-up of kidney function.

Another method to derive AKI subphenotypes is to use unsupervised clustering analyses, such as LCA. Bhatraju and colleagues applied LCA to a panel of 29 different variables in two cohorts of critically ill patients with AKI. They identified two AKI subphenotypes (AKI-SP1 and AKI-SP2) with different clinical characteristics and associations with clinical outcomes, even after adjusting for both illness and AKI severity [8]. They also found heterogeneity in treatment effect in a post-hoc analysis of patients with sepsis-associated AKI from the Vasopressin and Septic Shock Trial (VASST) trial, patients with AKI-SP1 had lower mortality with the early addition of vasopressin with norepinephrine therapy, while patients with AKI-SP2 had no difference in mortality [8, 38]. These findings are in contrast to the overall results in the VASST trial that demonstrated no mortality benefit with the early addition of vasopressin therapy for the treatment of shock. The findings also highlight the importance of identifying biologically distinct AKI subphenotypes as they may respond differently to treatments in clinical trials. Other research groups have also applied LCA to ICU cohorts with AKI and have identified two AKI subphenotypes. Wiersema and colleagues studied 301 patients with sepsis-associated AKI and applied LCA to 30 different variables including 12 variables involved in systemic inflammation and endothelial dysfunction [10]. They identified two AKI subphenotypes with differing clinical characteristics and outcomes [10].

Heterogeneity in the AKI clinical syndrome may also limit the identification of novel mechanisms and genetic risk for AKI. A systematic review in 2009 concluded that genetic studies in AKI have been inconsistent and contradictory [39]. One reason has been the lack of consensus on defining AKI by the time of that report. For example, the authors found five different definitions of AKI used in prior genetic studies. While the KDIGO definition of AKI is now widely used, AKI remains as a syndromic diagnosis that may be too heterogeneous to allow the identification of genetic risk factors. Thus, leveraging AKI subphenotypes may overcome the heterogeneity in the AKI clinical definition. Bhatraju et al. leveraged the previously described AKI subphenotypes to evaluate genetic risk in the development of AKI [40]. They performed a targeted genetic study to identify single nucleotide polymorphisms (SNPs) within 50 kb of the *ANGPT1, ANGPT2* and *TNFRSF1A* genes associated with AKI- SP2 in 452 subjects. They demonstrated that a SNP (rs2920656) near *ANGPT2* was associated with reduced risk for AKI-SP2 and this SNP was associated with decreased plasma concentrations of angiopoietin-2 (Ang-2). These findings support the pathophysiologic role of Ang-2 in AKI, also as a therapeutic target. Moreover, genetic susceptibilities may be concentrated in certain populations, such as another genetic polymorphism related to increased Ang-2 concentrations in septic ARDS in subjects with European ancestry [41]. A number of studies in other fields, such as diabetes mellitus [42] and asthma [43] have also leveraged disease subphenotypes to discover novel genetic variants associated with disease.

The work completed to date in identifying biomarker-based AKI subphenotypes raises the question of whether these AKI subphenotypes are specific to AKI or are found in other clinical syndromes, ARDS or sepsis. As in ARDS, sepsis and other diseases, circulating biomarkers of endothelial activation and inflammation are relevant and not specific to AKI. These findings imply potential parallels between critical illness syndromes and shared pathophysiological mechanisms across diseases. Some researchers have proposed transitioning from a disease specific model to identify subphenotypes to a ‘treatable traits’ model across diseases [44]. Potential examples in cancer include immunomodulatory therapy not specific to one type of cancer but effective in multiple types of cancer with high programmed death ligand-1 expression on tumor cells [45–47]. Another example is the use of mepolizumab, a monoclonal antibody that blocks interleukin-5 signaling, in eosinophilic lung disease irrespective if patients have asthma or chronic obstructive pulmonary disease [48, 49].

## Subphenotypes of other ICU syndromes

Analogous to AKI, there has been tremendous interest in defining subtypes of other forms of critical illness, including sepsis and the ARDS [16]. In the context of ARDS, two subphenotypes can reliably be identified in clinical trial populations. The hyperinflammatory subphenotype is characterized by higher levels of pro-inflammatory biomarkers including interleukin-6, interleukin-8, soluble tumor necrosis factor receptor-1, and plasminogen activator inhibitor-1 and higher mortality [50]. In contrast, the hypoinflammatory subphenotype is associated with higher levels of protein C and bicarbonate, as well as higher systolic blood pressure. Patients with the hyperinflammatory and hypoinflammatory subphenotypes have differential responses to a number of therapies, including fluid management, positive end-expiratory pressure, and statins [50–52]. For example, in the re-analysis of HARP-2 trial, there was improvement in survival with simvastatin therapy in patients with the hyper-inflammatory subphenotype [52]. These two subphenotypes are also identifiable in more generalizable, prospective observational cohort studies [53]. Finally, these subphenotypes can be reliably identified using a parsimonious subset of three biomarkers, raising the possibility of near real-time point-of-care biomarker measurement and predictive enrichment for clinical trials [54].

In an analysis using only biomarkers to identify subphenotypes, “uninflamed” and “reactive” subphenotypes were identified and linked to a number of signaling pathways in whole blood transcriptomic studies [55]. These subphenotypes are characterized by many of the same biomarkers as the “hyperinflammatory” and “hypoinflammatory” subphenotypes and emphasize the concept that a hyperinflammatory state is associated with adverse outcomes across a wide variety of critical illnesses and can be used to target specific therapeutic interventions in clinical trials.

Subphenotypes of sepsis have been studies in both children and adults. Here, the number of subphenotypes has varied more than in studies of ARDS, perhaps due to the larger overall populations studied and therefore the ability to derive more subphenotypes. Nonetheless, a number of important themes emerge from these studies. First, due to the large number of patients admitted with sepsis, it is possible to perform very large, clinical subphenotyping studies using data from the electronic health record. Some of these studies have focused on the trajectory of a limited number of variables (for example, temperature) [56], whereas others have focused on clustering patients based on data available at the time of presentation [57, 58]. These subphenotypes vary with regards to clinical characteristics and mortality; however, these subphenotypes may not add significantly to our biological insights regarding sepsis. In some cases, subsequent analyses have linked the inflammatory response to clinical subphenotypes, including the temperature-based subphenotypes [59]. Second, subphenotypes with variable response to therapies can be identified in children and adults. For example, re-analysis of clinical trials of an interleukin-1 receptor antagonist demonstrate differential outcomes by subphenotype [60, 61]. However, these studies also highlight the importance of studying both children and adults and the need for replication. That is, in analyses of the impact of steroids on outcomes in sepsis, although one pediatric subclass had higher mortality with corticosteroid therapy, but the adults did not [62, 63]. More work is clearly needed to better define and understand sepsis subphenotypes, which may have additional complexities due to underlying comorbidities (e.g., immunosuppressed states) that increase the risk of infection and may alter the host response to infection as well. Finally, a number of these studies have linked sepsis subphenotypes to gene expression patterns and host response, which will be critical to identifying precision therapies for sepsis. However, more work will clearly be needed to define subtypes within this complex syndrome [64].

## Current and future implications

To date, the vast majority of AKI subphenotyping work has focused on differences in prognosis -specifically, identifying which patients with AKI are likely to suffer poor outcomes, including death. As such, the current implications of this work, if validated and applied clinically, are largely based in *prognostic enrichment*. For example, several groups have now identified clinical and/or biomarker-based subphenotypes of sepsis-associated AKI in both adults [7, 8, 10, 65] and children [66, 67] that are associated with various outcomes of interest, including increased likelihood of requiring KRT, renal non-recovery, and mortality. These subphenotyping strategies could be applied at the bedside to inform clinical care (i.e. earlier consideration of KRT in high-risk patients), and perhaps more importantly, to guide risk-informed enrollment of patients into future therapeutic trials (i.e. to enroll only patients at high risk for KRT in trials examining optimal timing of initiation). Unfortunately, most of these tools have thus far failed to translate to the bedside, likely due to a combination of lack of largescale validation [7, 8, 10, 65–67], issues with timely availability of included biomarkers [8, 10, 67], genetic testing and overall complexity of the subphenotyping models [10, 65]. These are all issues that will need to be addressed in order to make real-time subphenotyping of AKI for prognostic enrichment a reality. Regarding the identification of ARDS subphenotypes, on-going projects are already searching solutions for feasible bedside identification of subphenotypes using machine-learning [68] or point-of-care biomarker assays (NCT04009330).

While prognostic enrichment is an important component of a personalized approach to AKI management, the ultimate goal of subphenotyping any heterogeneous disorder is to identify and employ precision therapeutics (i.e. *predictive enrichment*). While predictive enrichment strategies for AKI remain limited, recent AKI subphenotyping work has highlighted the potential for precision vasoactive selection. The application of two unique AKI subphenotypes (AKI-SP1 and AKI-SP2) in a subset of patients from the VASST trial demonstrated that patients classified as AKI-SP1—characterized by lesser degrees of endothelial activation and inflammation than AKI-SP2—had improved 28- and 90-day mortality when they received vasopressin compared to norepinephrine, though rates of renal recovery did not differ [4]. While direct links between the underlying biology of AKI-SP1 patients and their response to vasopressin have not been made, continued molecular subphenotyping of AKI using strategies such as these is required to identify future predictive enrichment targets and develop novel therapeutics. Unfortunately, similar to subphenotyping for prognostic enrichment, significant work needs to be done to translate these tools to the bedside of patients. In particular, the ability to rapidly subphenotype a patient with AKI remains the most significant barrier, given that many of the patients who would benefit most from this care are critically ill with evolving pathology and require time-sensitive decision making.

## Conclusions

Subphenotyping helps to differentiate patients with differing pathophysiologic mechanisms, severity of illness, and outcome amongst all patients with AKI. Clustering analyses including data from not routinely measured biomarkers have revealed subphenotypes with potentially distinct pathophysiology regarding response to inflammation thus opening avenues to research of targeted therapies. More research to validate the discovered AKI subphenotypes and to develop methods to distinct various subphenotypes rapidly at the bedside are needed.

## Data Availability

Not applicable.

## Abbreviations

AKI: Acute kidney injury
AKI-SP: Acute kidney injury subphenotype
Ang-2: Angiopoietin-2
ARDS: Acute respiratory distress syndrome
CCL14: C–C motif chemokine ligand 14
ICU: Intensive care unit
IGFBP7: Insulin-like growth factor-binding protein 7
KIM-1: Kidney injury molecule 1
KDIGO: Kidney diseases improving global outcomes
KRT: Kidney replacement therapy
LCA: Latent class analysis
NGAL: Neutrophil gelatinase-associated lipocalin
SNP: Single nucleotide polymorphisms
TIMP-2: Tissue inhibitor of metalloproteinase-2
VASST: Vasopressin and Septic Shock Trial
